# Analysis of pathogen distribution and association with lipid levels in patients with diabetic foot

**DOI:** 10.3389/fmed.2026.1799737

**Published:** 2026-05-22

**Authors:** Yan Chen, Weihong Chen, Yinxia Zhuo

**Affiliations:** 1 Department of Endocrinology, Ma’anshan People’s Hospital, Ma’anshan, Anhui, China; 2 Department of Health Management Center, Ma’anshan People’s Hospital, Ma’anshan, Anhui, China

**Keywords:** antimicrobial susceptibility, diabetic foot, lipid level, pathogen, personalized treatment

## Abstract

**Background:**

Diabetic foot infection (DFI) is a severe complication of diabetes, and dyslipidemia is common in affected patients. However, the relationship between lipid profiles, pathogen distribution, and antimicrobial resistance in DFI remains unclear.

**Methods:**

This retrospective study analyzed clinical and microbiological data from 85 patients with diabetic foot treated between January 2021 and August 2024. Lipid parameters (total cholesterol, triglycerides, HDL-C, and LDL-C) were recorded. Pathogens were identified using routine culture and biochemical methods, and antimicrobial susceptibility was assessed by disk diffusion according to CLSI guidelines.

**Results:**

Gram-positive bacteria predominated (54.12%), followed by gram-negative bacteria (38.82%), with *Staphylococcus aureus* and *Pseudomonas aeruginosa* being the most common isolates. Gram-positive bacteria showed the highest susceptibility to linezolid and vancomycin, whereas gram-negative bacteria were most sensitive to amikacin and piperacillin-tazobactam. No significant differences were observed in lipid levels among different pathogen groups, or between drug-resistant and non-resistant or multidrug-resistant and non-multidrug-resistant infections (all *p* > 0.05).

**Conclusion:**

Lipid profiles were not significantly associated with pathogen distribution or antimicrobial resistance patterns in patients with diabetic foot infection. Further prospective studies are needed to clarify the role of lipid metabolism in infection susceptibility and clinical outcomes.

## Introduction

Diabetic foot is a serious complication of diabetes and is associated with substantial morbidity, mortality, and impaired quality of life (1, 2). It commonly develops in the setting of peripheral neuropathy and peripheral vascular disease, which predispose patients to foot ulceration and tissue damage (3). Once ulceration occurs, secondary infection may further aggravate tissue destruction and increase the risk of delayed healing, hospitalization, and amputation. DFI is caused by a broad spectrum of microorganisms, predominantly gram-positive and gram-negative bacteria, with occasional fungal involvement (4). Because antimicrobial susceptibility varies substantially across pathogens, accurate microbiological characterization is essential for guiding appropriate antibiotic therapy and improving clinical outcomes (5).

The widespread use of broad-spectrum antibiotics has contributed to the emergence of resistant organisms, making the treatment of DFI increasingly challenging (6, 7). Moreover, the microbial profile of DFI may vary according to geographic, economic, and environmental factors (8, 9). Such heterogeneity complicates empirical antibiotic selection and underscores the importance of understanding local pathogen distribution and antimicrobial susceptibility patterns.

Dyslipidemia is a common metabolic abnormality in patients with diabetes and is closely related to the development and progression of diabetic complications (10). Previous studies suggest that dyslipidemia may indirectly influence the infection process in diabetic foot by impairing host immune function, promoting chronic inflammation, and worsening vascular dysfunction (11–13). These changes may alter the wound microenvironment and local host defense, thereby potentially affecting microbial colonization, persistence, and the distribution of dominant pathogens (14, 15). In addition, patients with metabolic dysregulation may be more likely to develop chronic or severe wounds requiring repeated antibiotic exposure, which could theoretically contribute to the selection of resistant organisms (16, 17). However, data on the relationship between systemic lipid levels and pathogen distribution or antimicrobial resistance patterns in DFI remain limited.

Therefore, this retrospective study analyzed the distribution of pathogens and their antimicrobial susceptibility patterns in patients with diabetic foot and further explored the association between lipid levels and pathogen distribution as well as resistance patterns. The aim was to provide microbiological evidence for local clinical practice and to offer preliminary insight into the potential relationship between lipid metabolism and infection characteristics in DFI.

## Methods

### Research subjects

**Figure 1:**
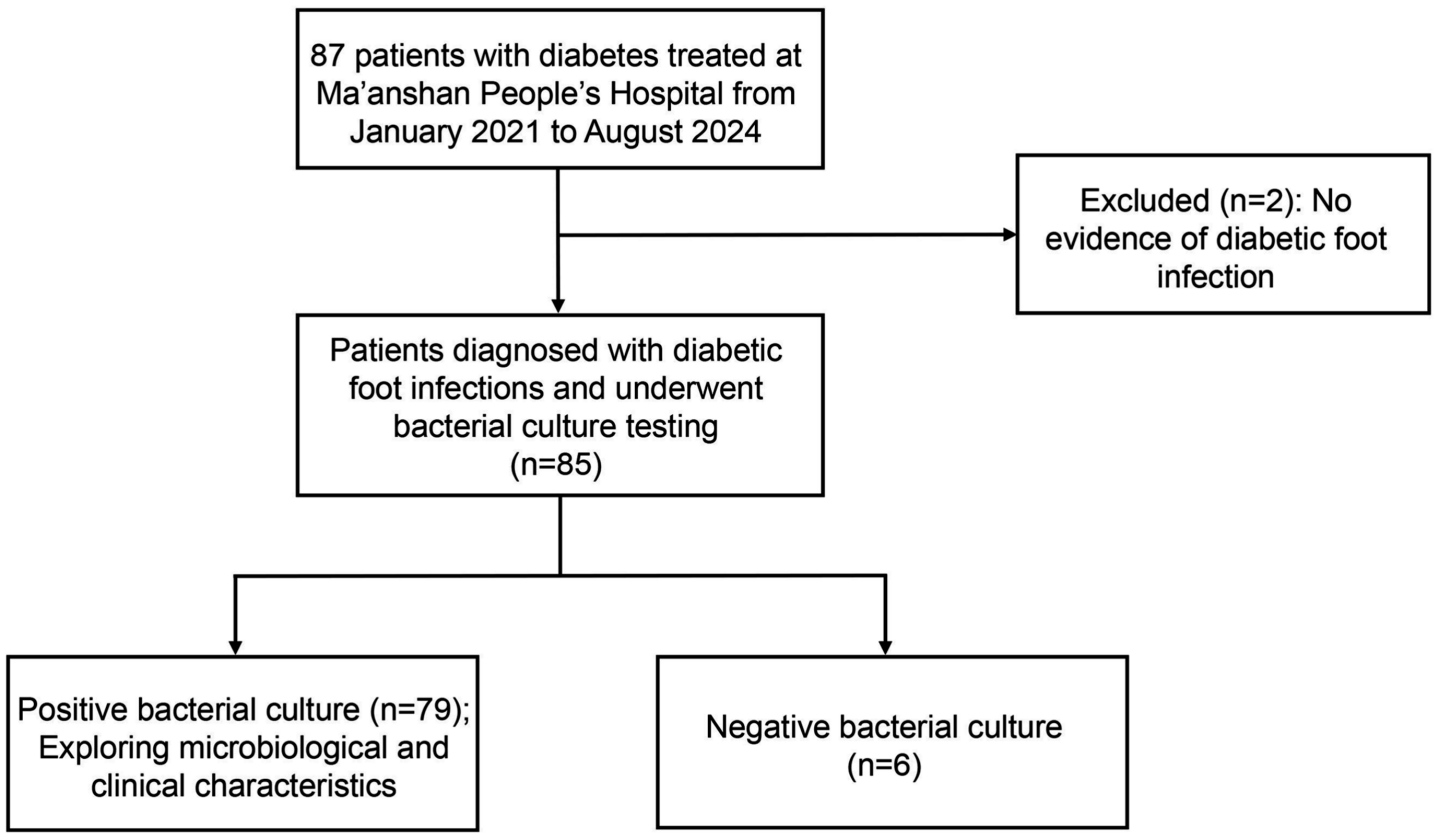
Flowchart of pathogen detection and inclusion for patients with diabetic foot.

This study retrospectively analyzed the clinical data of 85 patients with diabetic foot treated at Ma’anshan People’s Hospital between January 2021 and August 2024, and the study flow is shown in Figure 1. Inclusion criteria were as follows: (1) patients were diagnosed with diabetes and had foot ulcers; and (2) complete clinical data were available. The diagnosis of diabetic foot infection (DFI) was established according to the criteria of the International Working Group on the Diabetic Foot/Infectious Diseases Society of America (IWGDF/IDSA) guidelines (18), which define infection as the presence of at least two classical signs of inflammation (e.g., erythema, warmth, swelling, tenderness, or purulent discharge).

The study exclusion criteria were as follows: (1) in the acute phase of cardiovascular or cerebrovascular events; (2) with severe liver or kidney injury; (3) with neurological diseases or infectious diseases of other systems; (4) had incomplete treatment records. The study was approved by the ethics committee of Ma’anshan People’s Hospital (Approval No: 2024-10-04) and was conducted in accordance with the *Declaration of Helsinki*. All patients signed informed consent.

### Collection of clinical data

Clinical data were obtained from patients’electronic medical records, including gender, age, duration of diabetes, duration of foot ulcers, body mass index (kg/m^2^), waist-to-hip ratio, Wagner-Meggit classification (19), classification according to the International Working Group on the Diabetic Foot/Infectious Diseases Society of America (18), blood pressure (systolic and diastolic pressure), lipid levels [total cholesterol (TC), triglycerides (TG), low-density lipoprotein cholesterol (LDL-C), high-density lipoprotein cholesterol (HDL-C)], glycated hemoglobin, inflammatory markers (white blood cell count, C-reactive protein), apolipoproteins [apolipoprotein AI (ApoAI), ApoB] and comorbidities such as hypertension, coronary artery disease, diabetic ketoacidosis, chronic kidney disease, and cerebrovascular diseases. Ulcer severity was assessed using the Wagner-Meggit classification system (19), a widely used grading system for diabetic foot lesions that classifies ulcers according to the extent of tissue involvement, infection, and gangrene. In this study, patients were categorized into Wagner grades 1–4.

Information on prior antibiotic exposure before sample collection was not consistently available due to the retrospective nature of the study and was therefore not included in the analysis.

### Microbiological identification

Before collecting samples, the skin around the ulcer was disinfected with povidone-iodine, followed by rinsing the ulcer with sterile saline and performing sharp debridement. Sample collection was conducted according to the Levine technique, where a sterile swab was used to rotate for 7 s within a 1 cm^2^ area at the base of the ulcer to collect exudate and tissue samples. Then, the samples were immediately placed in sterile containers and sent to the laboratory within 30 min.

Upon receipt, a direct smear of the original specimen was prepared for microscopic examination, including assessment of bacterial morphotypes and the presence of inflammatory cells, as part of the standard laboratory workflow. Samples were inoculated onto MacConkey agar, mannitol salt agar, and blood culture medium, followed by incubation at 37 °C for 24 h. After microbial growth was observed, Gram staining was performed to differentiate between Gram-positive and Gram-negative bacteria. Subsequently, all isolates underwent biochemical identification using standard methods. Gram-negative bacteria underwent further biochemical tests, including the oxidase test, hydrogen sulfide-indole-motility agar, urea agar, triple sugar iron agar, Simmons’ citrate agar tests, lysine iron agar, motility-indole-ornithine agar, the methyl red and Voges-Proskauer tests. Gram-positive bacteria underwent catalase, coagulase, and bile esculin tests. *Staphylococcus epidermidis* ATCC 35984 was used as the positive control, while *Staphylococcus epidermidis* ATCC 12228 served as the negative control (20, 21). Each bacterial isolate was tested in triplicate to ensure the reliability of the results, and the species were identified using the VITEK® 2 system (bioMérieux). To avoid duplication bias, repeated isolates from the same patient were excluded, and only the first isolate per patient was included in the analysis.

### Antimicrobial susceptibility analysis

The disk diffusion method was employed to evaluate the antimicrobial susceptibility of the identified pathogens to commonly used antibiotics (22). The drugs tested for gram-positive bacteria included oxacillin (1 μg), penicillin G (10 μg), piperacillin-tazobactam (100/10 μg), ampicillin (10 μg), cefotaxime (30 μg), ceftazidime (30 μg), ceftriaxone (30 μg), cefepime (30 μg), cefoperazone (30 μg), gentamicin (10 μg), erythromycin (15 μg), amikacin (30 μg), clindamycin (2 μg), azithromycin (15 μg), ciprofloxacin (5 μg), levofloxacin (5 μg), moxifloxacin (5 μg), tigecycline (15 μg), linezolid (30 μg), vancomycin (30 μg), and rifampin (5 μg). The drugs used for testing gram-negative bacteria included piperacillin-tazobactam, cefoperazone-sulbactam (75/30 μg), cefotaxime, cefepime, cefoxitin (30 μg), ceftriaxone, cefoperazone, ceftazidime, ertapenem (10 μg), aztreonam (30 μg), amikacin, gentamicin, levofloxacin, ciprofloxacin, and moxifloxacin. *Staphylococcus aureus* ATCC 43300 and *Escherichia coli* ATCC 25922 were used as control strains in the experiment (23). Antimicrobial susceptibility results were interpreted according to the Clinical and Laboratory Standards Institute (CLSI) M100 Performance Standards for Antimicrobial Susceptibility Testing (2023 edition). Multidrug-resistant (MDR) bacteria were defined according to the international expert consensus proposed by Magiorakos et al. (24), namely non-susceptibility to at least one agent in three or more antimicrobial categories. Although a broad antimicrobial panel was tested as part of routine laboratory practice, only agents with CLSI-approved interpretive criteria were included in the final analysis and reporting.

### Statistical analysis

All data were statistically analyzed using SPSS 22.0 software. Descriptive statistics for normally distributed data were presented as mean ± standard deviation (SD), while non-normally distributed data were presented as median (IQR). Categorical variables are expressed as frequencies and percentages. For comparisons between two groups, an independent samples *t*-test was applied for normally distributed data, and the Mann–Whitney U test was used for non-normally distributed data. A one-way analysis of variance (ANOVA) was employed for comparisons among three or more groups with normally distributed data, followed by Tukey’s *post hoc* test for pairwise comparisons. For non-normally distributed data, the Kruskal–Wallis test was used, followed by Dunn’s test for pairwise comparisons. Statistical significance was considered when *p* < 0.05.

## Results

### Baseline characteristics

A total of 85 patients were included, with a mean age of 62.34 ± 13.78 years (Table 1). Most patients were male (71.76%), and the mean duration of diabetes was 12.67 ± 7.78 years. Regarding ulcer characteristics, Wagner grade 2 was the most common category (65.88%).

**Table 1:** Baseline characteristics of patients with diabetic foot.

Variables	Results
Gender (%)	
Male	61 (71.76)
Female	24 (28.24)
Age (year)	62.34 ± 13.78
Duration of diabetes (year)	12.67 ± 7.78
Duration of diabetic foot (day)	58.53 ± 18.93
BMI (kg/m^2^)	20.90 ± 2.08
WHR	0.72 ± 0.07
Wagner-Meggit classification (%)	
Grade 1	8 (9.41)
Grade 2	56 (65.88)
Grade 3	7 (8.23)
Grade 4	14 (16.47)
SBP	129.76 ± 22.77
DBP	75.15 ± 12.10
TC (mmol/L)	3.73 ± 1.29
TG (mmol/L)	1.34 ± 1.07
HDL-C (mmol/L)	0.97 ± 0.30
LDL-C (mmol/L)	2.15 ± 0.88
HbA1c (%)	10.13 ± 2.68
White blood cell count (×10^9^/L)	3.20 ± 0.94
CRP (mg/L)	20.42 ± 8.21
ApoAI (g/L)	0.81 ± 0.24
ApoB (g/L)	0.76 ± 0.23
Lp-a (mg/L)	221.3 (272.05)
Hypertension (%)	
Without	65 (76.47)
With	20 (23.53)
CAD (%)	
Without	77 (90.59)
With	8 (9.41)
DKA (%)	
Without	80 (94.12)
With	5 (5.88)
CKD (%)	
Without	83 (97.65)
With	2 (2.35)
Cerebrovascular disease (%)	
Without	76 (89.41)
With	9 (10.59)

Data were presented as mean ± SD, median (IQR), or *n* (%). BMI, body mass index; WHR, waist-to-hip ratio; SBP, systolic blood pressure; DBP, diastolic blood pressure; TC, total cholesterol; TG, triglycerides; HDL-C, high-density lipoprotein cholesterol; LDL-C, low-density lipoprotein cholesterol; HbA1c, glycated hemoglobin; CRP, C-reactive protein; ApoAI, apolipoprotein AI; ApoB, apolipoprotein B; Lp-a, lipoprotein(a); CAD, coronary artery disease; DKA, diabetic ketoacidosis; CKD, chronic kidney disease.

The average systolic blood pressure (SBP) was 129.76 ± 22.77 mmHg, and diastolic blood pressure (DBP) was 75.15 ± 12.10 mmHg. Regarding lipid profiles, the average TC was 3.73 ± 1.29 mmol/L, TG was 1.34 ± 1.07 mmol/L, HDL-C was 0.97 ± 0.30 mmol/L, and LDL-C was 2.15 ± 0.88 mmol/L.

Among inflammatory markers, the average white blood cell count was 3.20 ± 0.94 × 10^9^/L, CRP was 20.42 ± 8.21 mg/L, ApoAI was 0.81 ± 0.24 g/L, ApoB was 0.76 ± 0.23 g/L, and lipoprotein(a) level was 221.3 (272.05) mg/L.

In terms of comorbidities, 23.53% of patients had hypertension, 9.41% had coronary artery disease (CAD), 5.88% had diabetic ketoacidosis (DKA), 2.35% had chronic kidney disease (CKD), and 10.59% had cerebrovascular disease.

### Microbial identification

Among the 85 patients, 46 (54.12%) had gram-positive bacteria, 33 (38.82%) had gram-negative bacteria, and no bacteria or fungi were detected in 6 patients (Table 2). The most prevalent gram-positive bacteria were *Staphylococcus aureus* (31.76%), *Streptococcus agalactiae* (5.88%), and *Streptococcus pyogenes* (4.71%). Gram-negative bacteria were predominantly *Pseudomonas aeruginosa* (7.06%), followed by *Klebsiella pneumoniae* (4.71%) and *Escherichia coli* (3.53%).

**Table 2:** Microbial identification.

Type of microorganism	*n* (%)
Gram-positive bacteria	46 (54.12)
*Staphylococcus aureus*	27 (31.76)
*Staphylococcus epidermidis*	3 (3.53)
*Staphylococcus simulans*	1 (1.18)
*Staphylococcus warneri*	1 (1.18)
*Staphylococcus hemolyticus*	1 (1.18)
*Streptococcus pyogenes*	4 (4.71)
*Streptococcus agalactiae*	5 (5.88)
*Streptococcus anginosus*	1 (1.18)
*Corynebacterium* spp.	2 (2.35)
*Enterococcus faecalis*	1 (1.18)
Gram-negative bacteria	33 (38.82)
*Proteus vulgaris*	2 (2.35)
*Burkholderia cepacia*	1 (1.18)
*Escherichia coli*	3 (3.53)
*Pseudomonas aeruginosa*	6 (7.06)
*Klebsiella pneumoniae*	4 (4.71)
*Klebsiella oxytoca*	2 (2.35)
*Proteus mirabilis*	1 (1.18)
*Morganella morganii*	1 (1.18)
*Raoultella planticola*	1 (1.18)
*Raoultella ornithinolytica*	1 (1.18)
*Serratia marcescens* complex	1 (1.18)
*Enterobacter cloacae* complex	2 (2.35)
*Providencia stuartii*	1 (1.18)
*Eikenella corrodens*	1 (1.18)
*Acinetobacter haemolyticus*	1 (1.18)
*Moraxella catarrhalis*	1 (1.18)
*Citrobacter* spp.	4 (4.71)
No bacteria or fungi detected	6 (7.06)

Data were presented as *n* (%).

### Antimicrobial susceptibility

Analysis of antimicrobial susceptibility showed that gram-positive bacteria had the highest susceptibility to gentamicin, linezolid, and vancomycin (all 80.43%), followed by levofloxacin (74.47%), ciprofloxacin (65.96%), and rifampicin (68.09%). Only antibiotics with CLSI-approved breakpoints were included for gram-positive organisms; therefore, agents such as cefotaxime, cefoperazone, and azithromycin were not analyzed.

Among gram-negative bacteria, piperacillin-tazobactam (71.88%), amikacin (71.88%), ceftazidime (68.75%), and cefepime (65.62%) showed the highest susceptibility, followed by levofloxacin (50.00%) and ciprofloxacin (46.88%). Agents lacking CLSI breakpoints for gram-negative organisms, including penicillin G, cefoperazone, azithromycin, tigecycline, and vancomycin, were excluded from analysis (Table 3).

**Table 3:** Antimicrobial susceptibility (CLSI-compliant).

Antimicrobial drug	Gram-positive bacteria (*n* = 46)	Gram-negative bacteria (*n* = 33)
Oxacillin	19 (41.30)	N/A
Penicillin G	2 (4.35)	N/A
Ampicillin	9 (19.57)	N/A
Gentamicin	37 (80.43)	20 (60.61)
Erythromycin	20 (43.48)	N/A
Clindamycin	29 (63.04)	N/A
Ciprofloxacin	31 (67.39)	15 (45.45)
Levofloxacin	35 (76.09)	16 (48.48)
Moxifloxacin	27 (58.70)	N/A
Linezolid	37 (80.43)	N/A
Vancomycin	37 (80.43)	N/A
Rifampicin	32 (69.57)	N/A
Piperacillin-tazobactam	N/A	23 (69.70)
Ceftazidime	N/A	22 (66.67)
Ceftriaxone	N/A	15 (45.45)
Cefepime	N/A	21 (63.64)
Amikacin	N/A	23 (69.70)
Ertapenem	N/A	21 (63.64)
Aztreonam	N/A	16 (48.48)

Data were presented as *n* (%). “N/A” indicates that the drug is not routinely used or interpreted for that bacterial group according to CLSI guidelines.

The top four gram-positive bacteria were *Staphylococcus* spp., *Streptococcus* spp., *Corynebacterium* spp., and *Enterococcus* spp., with antimicrobial susceptibility shown in Table 4. *Staphylococcus* spp. showed the highest susceptibility to gentamicin (96.97%), followed by linezolid and vancomycin (96.97%). High susceptibility was also observed for rifampicin (96.97%) and levofloxacin (84.85%). For *Streptococcus* spp., susceptibility was mainly observed to penicillin G and ampicillin, with lower susceptibility to fluoroquinolones. *Corynebacterium* spp. demonstrated susceptibility primarily to linezolid and vancomycin, while susceptibility to fluoroquinolones was variable. *Enterococcus* spp. was susceptible to penicillin G, ampicillin, vancomycin, linezolid, and levofloxacin, with limited susceptibility to other β-lactam agents.

**Table 4:** Antimicrobial susceptibility of gram-positive bacteria.

Antimicrobial drug	*Staphylococcus* spp. (*n* = 33)	*Streptococcus* spp. (*n* = 10)	*Corynebacterium* spp. (*n* = 2)	*Enterococcus* spp. (*n* = 1)
Oxacillin	19 (57.58)	N/A	N/A	N/A
Penicillin G	0 (0.00)	1 (10.00)	N/A	1 (100)
Ampicillin	1 (3.03)	5 (50.00)	N/A	1 (100)
Erythromycin	19 (57.58)	1 (10.00)	N/A	N/A
Clindamycin	27 (81.82)	1 (10.00)	N/A	N/A
Gentamicin	32 (96.97)	N/A	N/A	N/A
Ciprofloxacin	26 (78.79)	N/A	0 (0.00)	1 (100)
Levofloxacin	28 (84.85)	3 (30.00)	0 (0.00)	1 (100)
Moxifloxacin	25 (75.76)	2 (20.00)	0 (0.00)	N/A
Linezolid	32 (96.97)	4 (40.00)	0 (0.00)	1 (100)
Vancomycin	32 (96.97)	4 (40.00)	0 (0.00)	1 (100)
Rifampicin	32 (96.97)	N/A	N/A	N/A

Data were presented as *n* (%). Only antibiotics with CLSI-approved interpretive criteria for each organism group are reported. N/A indicates no CLSI breakpoint is available.

The top four gram-negative bacteria were *Citrobacter* spp., *Pseudomonas* spp., *Klebsiella* spp., and *Proteus* spp., with antimicrobial susceptibility results summarized in Table 5. *Citrobacter* spp. demonstrated moderate susceptibility to multiple agents, including piperacillin-tazobactam and ceftazidime. In *Pseudomonas* spp., the highest susceptibility was observed for piperacillin-tazobactam, ceftazidime, cefepime, gentamicin, and amikacin, while susceptibility to fluoroquinolones was variable. *Klebsiella* spp. exhibited high susceptibility to third- and fourth-generation cephalosporins, aminoglycosides, and fluoroquinolones. *Proteus* spp. showed broad susceptibility across most tested agents, with lower susceptibility to fluoroquinolones.

**Table 5:** Antimicrobial susceptibility of gram-negative bacteria.

Antimicrobial drug	*Citrobacter* spp. (*n* = 4)	*Pseudomonas* spp. (*n* = 6)	*Klebsiella* spp. (*n* = 6)	*Proteus* spp. (*n* = 3)
Piperacillin-tazobactam	2 (50.00)	5 (83.33)	2 (33.33)	3 (100)
Ampicillin	1 (25.00)	N/A	0 (0.00)	0 (0.00)
Ceftazidime	2 (50.00)	4 (66.67)	6 (100.00)	3 (100)
Ceftriaxone	2 (50.00)	N/A	6 (100.00)	3 (100)
Cefepime	2 (50.00)	4 (66.67)	6 (100.00)	3 (100)
Gentamicin	2 (50.00)	4 (66.67)	6 (100.00)	3 (100)
Amikacin	2 (50.00)	4 (66.67)	6 (100.00)	3 (100)
Ciprofloxacin	2 (50.00)	3 (50.00)	6 (100.00)	1 (33.33)
Levofloxacin	2 (50.00)	3 (50.00)	6 (100.00)	3 (100)
Ertapenem	2 (50.00)	N/A	6 (100.00)	0 (0.00)
Aztreonam	2 (50.00)	1 (16.67)	6 (100.00)	0 (0.00)

Data were presented as n (%). Only antibiotics with CLSI-approved interpretive criteria for each organism group are reported. N/A indicates no CLSI breakpoint is available for that organism-drug combination.

### Correlation between lipid levels and bacterial infections

There were no significant differences in TC, TG, HDL-C, and LDL-C levels among patients with gram-positive bacteria, gram-negative bacteria, and those without detected pathogens (*p* > 0.05, Table 6). Similarly, no significant differences in lipid levels were observed among patients infected with *Staphylococcus* spp., *Streptococcus* spp., *Corynebacterium* spp., or *Enterococcus* spp. (*p* > 0.05, Table 7). No significant differences in lipid levels were observed among patients infected with *Citrobacter* spp., *Pseudomonas* spp., *Klebsiella* spp., or *Proteus* spp. (*p* > 0.05, Table 8).

**Table 6:** Comparison of lipid levels in patients with diabetic foot under different infection statuses.

Lipid level	Gram-positive bacteria (*n* = 46)	Gram-negative bacteria (*n* = 33)	No bacteria or fungi detected (*n* = 6)	*F*	*p*
TC	3.93 ± 1.38	3.52 ± 1.33	3.48 ± 1.04	0.924	0.402
TG	1.39 ± 1.22	1.23 ± 0.67	1.10 ± 0.75	0.368	0.694
HDL-C	1.08 ± 0.36	0.96 ± 0.37	1.03 ± 0.39	0.79	0.458
LDL-C	2.21 ± 0.84	2.25 ± 1.08	2.40 ± 1.03	0.155	0.857

Data were displayed as mean ± SD.

**Table 7:** Comparison of lipid levels among different genera of gram-positive bacteria.

Lipid level	*Staphylococcus* spp. (*n* = 33)	*Streptococcus* spp. (*n* = 10)	*Corynebacterium* spp. (*n* = 2)	*Enterococcus* spp. (*n* = 1)	*F*	*p*
TC	3.80 ± 1.06	4.26 ± 2.11	3.02 ± 0.22	4.46	0.638	0.595
TG	1.29 ± 0.94	2.10 ± 2.29	0.90 ± 0.32	0.50	1.206	0.319
HDL-C	1.05 ± 0.30	0.96 ± 0.32	0.83 ± 0.51	1.79	2.482	0.074
LDL-C	2.17 ± 0.79	2.05 ± 0.77	1.93 ± 0.66	2.54	1.141	0.935

Data were presented as mean ± SD or number (*n*).

**Table 8:** Comparison of lipid levels among different genera of gram-negative bacteria.

Lipid level	*Citrobacter* spp. (*n* = 4)	*Pseudomonas* spp. (*n* = 6)	*Klebsiella* spp. (*n* = 6)	*Proteus* spp. (*n* = 3)	*F*	*p*
TC	3.24 ± 1.06	3.77 ± 1.34	2.77 ± 0.33	4.60 ± 2.56	0.722	0.559
TG	1.05 ± 0.32	1.51 ± 1.07	1.13 ± 0.36	1.24 ± 0.68	0.296	0.828
HDL-C	0.83 ± 0.24	0.89 ± 0.30	1.00 ± 0.76	0.97 ± 1.14	0.602	0.627
LDL-C	1.96 ± 1.00	2.01 ± 0.94	2.36 ± 1.17	3.14 ± 2.25	1.165	0.918

Data were presented as mean ± SD.

### Relationship between drug resistance and lipid levels

Lastly, we compared the lipid levels between drug-resistant and non-resistant groups, as well as between multidrug-resistant and non-multidrug-resistant groups. No significant differences were observed in TC, TG, HDL-C, or LDL-C levels between the groups (*p* > 0.05, Tables 9, 10). The findings suggested that there may be no direct association between drug resistance and lipid levels in patients with diabetic foot.

**Table 9:** Comparison of lipid levels between drug-resistant and non-drug-resistant patients with diabetic foot.

Lipid level	Drug-resistant group (*n* = 49)	Non-resistant group (*n* = 36)	*t*	*p*
TC	3.95 ± 1.37	3.43 ± 1.10	1.871	0.065
TG	1.45 ± 1.30	1.17 ± 0.61	1.197	0.235
HDL-C	1.01 ± 0.29	0.92 ± 0.32	1.333	0.186
LDL-C	2.27 ± 0.87	2.01 ± 0.90	1.269	0.208

Data were presented as mean ± SD.

**Table 10:** Comparison of lipid levels between multidrug-resistant and non-multidrug-resistant patients with diabetic foot.

Lipid level	Non-multidrug-resistant group (n = 11)	Multidrug-resistant group (n = 38)	*t*	*p*
TC	3.65 ± 0.96	4.07 ± 1.47	0.832	0.410
TG	1.18 ± 0.41	1.54 ± 1.46	0.801	0.427
HDL-C	0.97 ± 0.31	1.02 ± 0.29	0.537	0.594
LDL-C	2.17 ± 0.82	2.29 ± 0.89	0.409	0.685

Data were presented as mean ± SD.

## Discussion

This study investigated the distribution of pathogens, their antimicrobial susceptibility profiles, and the relationship between lipid levels and infection types in patients with diabetic foot. Gram-positive bacteria predominated, with *Staphylococcus aureus* being the most frequently isolated species, followed by *Pseudomonas aeruginosa* among gram-negative bacteria. Antimicrobial susceptibility testing demonstrated high *in vitro* activity of linezolid and vancomycin against gram-positive isolates, whereas amikacin and piperacillin-tazobactam showed high susceptibility rates among gram-negative isolates. In accordance with CLSI guidelines, antibiotics without organism-specific interpretive criteria were excluded from susceptibility analysis, underscoring the importance of guideline-based interpretation in empirical antimicrobial selection.

Consistent with the findings of Liu et al. (25), gram-positive bacteria were the predominant pathogens in this cohort. However, unlike some reports from China describing a higher prevalence of gram-negative bacteria (26), this discrepancy may be influenced by differences in patient characteristics, wound severity, geographic location, and healthcare settings. As the primary pathogen in DFI (27), *Staphylococcus aureus* exhibited a high detection rate, which may be linked to the distinct pathogenic mechanisms and the immunodeficiency of patients with diabetes. Moreover, the hyperglycemic environment and immunodeficiency of patients with diabetes may provide more favorable conditions for the survival and spread of *Staphylococcus aureus* (28, 29). Additionally, this study identified other gram-positive bacteria, such as *Streptococcus agalactiae* and *Streptococcus pyogenes*. This finding indicated that various potential pathogens, apart from the primary ones, should be considered in DFI to ensure a thorough diagnosis. In addition, gram-negative bacteria such as *Pseudomonas aeruginosa* in DFI should not be overlooked, as they exhibit high virulence and antibiotic resistance, making treatment more complex (30, 31).

Although the average wound duration in this cohort was approximately 58 days, wound duration alone does not fully determine microbial composition. In this study, most ulcers were classified as Wagner grade 1–2, indicating that the majority of lesions were relatively less advanced in clinical severity. Even in chronic settings, less advanced diabetic foot ulcers are commonly dominated by gram-positive skin flora, particularly *Staphylococcus* species, whereas gram-negative and anaerobic organisms are more frequently associated with deep or extensively complicated wounds. In addition, wound specimens were collected using the Levine swab technique, which is widely used in routine clinical practice and is appropriate for less advanced or superficially accessible wounds. However, this approach may have limited sensitivity for detecting deeper tissue pathogens or mixed infections typical of advanced chronic wounds. Furthermore, as this was a retrospective study based on routine clinical microbiology records, patient-level characterization of polymicrobial infection was not feasible, which may have led to an underestimation of microbial complexity. Together, these clinical and methodological factors may explain the predominance of gram-positive bacteria observed in this cohort.

No bacteria or fungi were detected in six patients (7.06%) in this cohort. This finding may reflect limitations of conventional culture-based methods, which may fail to identify fastidious, low-abundance, or previously treated microorganisms. In addition, some patients with diabetic foot ulcers may present with culture-negative lesions, in which local inflammation can be influenced by non-infectious factors such as microvascular dysfunction or diabetic neuropathy (32). For cases where no pathogen was detected, clinical evaluation should be based on a comprehensive assessment of patient history, clinical symptoms, and laboratory findings. If necessary, advanced techniques such as high-throughput sequencing may be employed to provide a more thorough microbial identification and assist in clinical decision-making.

In terms of antimicrobial susceptibility, linezolid and vancomycin showed high *in vitro* activity against gram-positive isolates, whereas amikacin and piperacillin-tazobactam demonstrated high susceptibility rates among gram-negative isolates. Vancomycin, a glycopeptide antibiotic, and linezolid, an oxazolidinone, are widely used in the management of serious gram-positive infections and exhibited favorable susceptibility profiles in this study (33). In addition, gentamicin and levofloxacin showed relatively good *in vitro* activity, particularly against isolates with resistance to multiple antibiotic classes (34). Among gram-negative bacteria, amikacin, piperacillin-tazobactam, and ceftazidime demonstrated high susceptibility rates, supporting their potential utility in the treatment of diabetic foot infections caused by gram-negative pathogens. By contrast, cefoperazone, cefotaxime, and azithromycin showed limited in vitro activity against several bacterial species in this cohort where CLSI interpretive criteria were available, consistent with previous reports (35). These findings highlight the importance of susceptibility-guided antimicrobial selection to optimize treatment outcomes and avoid inappropriate empirical therapy.

In drug resistance testing, *Staphylococcus* spp. showed high susceptibility to oxacillin, rifampin, linezolid, and vancomycin. Oxacillin susceptibility suggests a predominance of methicillin-susceptible *Staphylococcus aureus* (MSSA) in this cohort, while linezolid and vancomycin remain important therapeutic options for infections caused by methicillin-resistant strains (36). Rifampin demonstrated good *in vitro* activity and is commonly used as part of combination regimens, particularly in biofilm-associated infections (37, 38). In contrast, *Staphylococcus* spp. exhibited low susceptibility to penicillin G and ampicillin in this study, suggesting limited effectiveness of these agents for empirical therapy. *Streptococcus* spp. showed high susceptibility to penicillin G and ampicillin but reduced susceptibility to certain cephalosporins, which may be associated with alterations in penicillin-binding proteins or other resistance mechanisms rather than *β*-lactamase production (39). Additionally, *Corynebacterium* spp. were relatively uncommon, and susceptibility interpretation was limited to antibiotics with available CLSI M45 breakpoints, primarily linezolid and vancomycin (40). *Enterococcus* spp. were infrequently isolated but clinically relevant due to their association with healthcare-associated infections and antimicrobial resistance (41). This study found that *Enterococcus* spp. exhibited susceptibility to penicillin G and ampicillin; however, interpretation should be made with caution, given the small number of isolates.

*Citrobacter* spp. are opportunistic pathogens widely distributed in the environment and have been reported to account for a proportion of diabetic foot infections (42, 43). In this study, *Citrobacter* spp. showed high susceptibility to piperacillin-tazobactam and ceftazidime, whereas very low susceptibility was observed for erythromycin and penicillin G, antibiotics not routinely recommended for Enterobacterales, indicating their limited clinical utility in Citrobacter-associated DFI. *Pseudomonas* spp., characterized by complex and variable resistance patterns, were highly susceptible to amikacin and gentamicin but showed poor susceptibility to several cephalosporins and tigecycline, consistent with previous reports (44). *Klebsiella* spp., associated with both hospital- and community-acquired infections (45), demonstrated moderate susceptibility to multiple agents, particularly ertapenem and ceftazidime. *Proteus* spp., another common gram-negative pathogen in DFI (46), showed high susceptibility to piperacillin-tazobactam, amikacin, and ertapenem but reduced susceptibility to several cephalosporins and tigecycline, underscoring the importance of susceptibility-guided individualized therapy.

In the present study, lipid profiles did not differ significantly among patients with gram-positive, gram-negative, or culture-negative diabetic foot infections, with comparable levels of TC, TG, HDL-C, and LDL-C observed across groups. These findings indicate that, within this cohort, baseline lipid levels were not strongly associated with infection type. Although dyslipidemia is common in patients with diabetes, its role in determining pathogen distribution in diabetic foot infections remains unclear. The development and progression of DFI are more likely influenced by factors such as host immune status, local perfusion, tissue injury severity, and bacterial virulence rather than lipid metabolism alone (47). Emerging experimental evidence suggests that host lipid metabolism may influence bacterial virulence and biofilm formation; however, these mechanisms have primarily been demonstrated in *in vitro* or preclinical models and were not evaluated in the present clinical study. Moreover, no significant differences in lipid parameters were observed between drug-resistant and non-resistant infections or between multidrug-resistant and non-multidrug-resistant groups. While these results suggest no evident association between lipid levels and antimicrobial resistance patterns in this cohort, they should be interpreted cautiously given the retrospective design and limited sample size.

Despite these findings, several limitations should be considered when interpreting the results. First, the retrospective design introduces inherent risks of incomplete data capture and potential selection bias, which may limit generalizability. Second, the relatively small sample size reduces statistical power, increasing the likelihood of type II error and potentially obscuring modest associations between lipid parameters and infection characteristics. Third, the microbiological data were derived from routine clinical cultures and were not structured for detailed patient-level characterization of infection complexity. As a result, differentiation between monomicrobial and polymicrobial infections was limited, and the true microbial burden of chronic wounds may have been underestimated. In addition, culture-based methods may preferentially detect readily cultivable organisms and may be influenced by prior antibiotic exposure, further affecting pathogen distribution. Moreover, anaerobic culture was not routinely performed, which may have led to underdetection of anaerobic organisms, particularly in chronic or more complex wounds. Fourth, vascular status was not systematically evaluated. Given the central role of peripheral arterial disease and local ischemia in diabetic foot pathophysiology, the absence of objective vascular assessments limits the interpretation of host-pathogen interactions. Fifth, potential confounding factors, including prior medication use, lipid-lowering therapy, and lifestyle-related variables, were not accounted for, and no multivariable adjustment was performed. Therefore, the observed lack of association between lipid levels and infection characteristics should be interpreted with caution, as residual confounding cannot be excluded. Lastly, lipid measurements were assessed at a single time point, and temporal changes were not evaluated, which may limit insight into dynamic metabolic-infectious interactions. Future prospective studies incorporating standardized microbiological sampling, vascular assessment, and multivariable analytical models are needed to more robustly define the relationship between lipid metabolism and infection patterns in diabetic foot.

## Conclusion

This study characterized pathogen distribution and antimicrobial susceptibility patterns in patients with diabetic foot and examined their association with lipid profiles. Gram-positive bacteria predominated, with distinct susceptibility patterns observed between gram-positive and gram-negative organisms. No significant associations were identified between lipid levels and pathogen type or antimicrobial resistance, suggesting that lipid metabolism was not associated with microbial distribution or resistance patterns in this retrospective cohort. Given the multifactorial nature of diabetic foot infections, future prospective studies with larger sample sizes and mechanistic designs are needed to further elucidate the role of metabolic factors in infection susceptibility and clinical outcomes.

## Data Availability

The original contributions presented in the study are included in the article/supplementary material, further inquiries can be directed to the corresponding author.
